# Silencing of a *Nicotiana benthamiana* ascorbate oxidase gene reveals its involvement in resistance against cucumber mosaic virus

**DOI:** 10.1007/s00425-023-04313-x

**Published:** 2024-01-16

**Authors:** Reshma Ahmed, Athanasios Kaldis, Andreas Voloudakis

**Affiliations:** 1 Laboratory of Plant Breeding and Biometry, Department of Crop Science, Agricultural University of Athens, 11855 Athens, Greece; 2 Department of Agricultural Biotechnology, Assam Agricultural University, Jorhat, Assam 785013 India

**Keywords:** CMV, Plant defense, Plant-virus interaction, Resistance genes, Reverse genetics, Tobacco

## Abstract

**Main conclusion:**

Silencing of an ascorbate oxidase (*AO*) gene in *N. benthamiana* enhanced disease severity from cucumber mosaic virus (CMV), showing higher accumulation and expansion of the spreading area of CMV.

**Abstract:**

A *Nicotiana benthamiana* ascorbate oxidase (*NbAO*) gene was found to be induced upon cucumber mosaic virus (CMV) infection. Virus-induced gene silencing (VIGS) was employed to elucidate the function of AO in *N. benthamiana*. The tobacco rattle virus (TRV)-mediated VIGS resulted in an efficient silencing of the *NbAO* gene, i.e., 97.5% and 78.8% in relative quantification as compared to the control groups (TRV::eGFP- and the mock-inoculated plants), respectively. In addition, AO enzymatic activity decreased in the TRV::NtAO-silenced plants as compared to control. TRV::NtAO-mediated *NbAO* silencing induced a greater reduction in plant height by 15.2% upon CMV infection. CMV titer at 3 dpi was increased in the systemic leaves of *NbAO*-silenced plants (a 35-fold change difference as compared to the TRV::eGFP-treated group). Interestingly, CMV and TRV titers vary in different parts of systemically infected *N. benthamiana* leaves. In TRV::eGFP-treated plants, CMV accumulated only at the top half of the leaf, whereas the bottom half of the leaf was “occupied” by TRV. In contrast, in the *NbAO*-silenced plants, CMV accumulated in both the top and the bottom half of the leaf, suggesting that the silencing of the *NbAO* gene resulted in the expansion of the spreading area of CMV. Our data suggest that the *AO* gene might function as a resistant factor against CMV infection in *N. benthamiana*.

**Supplementary Information:**

The online version contains supplementary material available at 10.1007/s00425-023-04313-x.

## Introduction

To defend against viral pathogens, plants trigger the post-transcriptional gene silencing (PTGS) mechanism which results in the generation of small interfering RNAs (siRNAs) that are highly specific to the target sequence of the invading virus. PTGS, also referred to as RNA silencing or RNA interference (RNAi), has been extensively studied in plants (Lindbo 2012; Tan et al. 2020). Initially, with the help of RNA-dependent RNA polymerase during viral replication, viral double-stranded RNA (dsRNA) is produced inside plant cells and is recognized by the dicer-like (DCL) proteins. DCLs are ribonucleases that cleave the viral dsRNA leading to the production of siRNAs which subsequently bind to the RNA-induced silencing complex (RISC) and execute the slicing of the viral RNAs in a homology-dependent manner (Waterhouse et al. 2001).

This highly specific gene silencing strategy has been exploited by scientists as a plant biotechnology tool—referred to as virus-induced gene silencing (VIGS)—in order to silence endogenous target genes of interest. To achieve the silencing of a gene in a plant, a fragment of the target sequence is cloned into a viral vector and then is introduced into the plant, usually via Agrobacterium-mediated plant transformation (Ruiz et al. 1998; Baulcombe 1999). During the *in planta* replication of the engineered virus, the activation of VIGS leads to the generation of siRNAs that prevent the expression or translation of the endogenous target gene, resulting in a significant knock-down (Senthil-Kumar and Mysore 2014). VIGS can be successfully applied for both forward and reverse genetic studies for the identification or the functional characterization of particular genes (Baulcombe 1999; Lu et al. 2003; Senthil-Kumar and Mysore 2011; Rojas et al. 2012). On the other hand, one limitation of the VIGS protocol could be the non-uniform silencing of the targeted genes in several areas of the plant body (Burch-Smith et al. 2004). VIGS protocol mainly includes tobacco rattle virus (TRV)-based vectors that are widely used in many plant species. Successful silencing of host genes using TRV has been done in most of the Solanaceous crops including tobacco (Ratcliff et al. 2001; Caplan et al. 2008), potato (Brigneti et al. 2004), tomato (Ekengren et al. 2003; Fu et al. 2005), petunia (Chen et al. 2004), and pepper (Chung et al. 2004). VIGS has also been applied in the model plant *Arabidopsis thaliana* (Cai et al. 2006; Pflieger et al. 2008) as well as in monocot species such as maize, rice, and barley (Scofield et al. 2005; Ding et al. 2006).

Ascorbate oxidases (AO) are glycoproteins that fall under the family of blue copper oxidase enzymes, are mainly present at the peripheral region of the cell wall close to the plasma membrane, and are encoded by a multigene family (De Tullio et al. 2013; Kumari et al. 2016). Interestingly, these enzymes are plant- and fungal-specific, are involved in cell wall modification, and catalyze oxygen reduction to water by employing ascorbic acid (AA) as the electron donor, having as a result an antioxidant function (De Tullio et al. 2013). Furthermore, it is reported that AO proteins, through the oxidation of AA, play a vital role in the defense system of plants by controlling the ascorbate/dehydroascorbate (DHA) ratio in the apoplast (Singh et al. 2021). This modulation of apoplastic redox status is proposed as the cue for triggering plant responses to biotic stresses (Pignocchi and Foyer 2003). Moreover, it is now considered that the complex role of oxidants and antioxidants constitute an efficient way to combine various cues and induce both local and systemic responses to stresses (Mittler et al. 2011). The induction of AO enzyme has been reported under oxidative stress (Sanmartin et al. 2003) and its crucial participation in the regulation of oxidative stress has been shown (De Tullio 2010).

Plant–virus interaction represents a complex process with many plant factors involved. Virus infection enhances the expression of several plant genes that alter biosynthetic pathways in response to viral progeny production whereas host defense genes could be activated in order to stop the infection (Šubr et al. 2020). Several studies suggest the involvement of AOs in plant–viral interactions. In a RNAseq analysis performed in *N. tabacum-*tobacco mosaic virus (TMV) susceptible interaction, two *AO* genes (encoding for NtAO_A0A1S3Y315 and NtAO_A0A1S3ZI67) were found to be induced early in the infection process (Voloudakis’ lab unpublished data). Kumari et al. (2016) found that an *NbAO* (Niben101Scf03483g00002.1) is involved in CMV infection in *Nicotiana benthamiana*. Interestingly, in the same study, it was demonstrated that an AO protein from *Cucumis sativus* (CsAO4) binds to the movement protein of cucumber mosaic virus (CMV). Furthermore, Wu et al. (2017) demonstrated that the induction of an *AO* gene in rice constitutes a basic component of the defense system of the plants against rice stripe virus (RSV) and rice black streaked dwarf virus (RBSDV).

In the present study, we aimed at understanding the function of an *AO* gene in response to CMV infection in *N. benthamiana* employing a TRV-mediated VIGS approach. As a model plant, *N. benthamiana* has been utilized globally in plant virology because of its evident role in hyper-susceptibility to virus infections (Wylie et al. 2015). CMV infects more than 1200 species in 100 plant families including both dicots and monocots. CMV is a single-stranded positive-sense RNA virus that belongs to the genus Cucumovirus, family Bromoviridae. CMV genome consists of RNAs 1–3, with RNA1 encoding the viral helicase, RNA2 encoding the viral replicase, and RNA3 encoding the viral movement protein 3a. Two sub-genomic RNAs are produced, namely RNA4 (derived from RNA3) encoding the coat protein and RNA4A (derived from RNA2) encoding the 2b RNA silencing suppressor which potentiates the cell-to-cell and the long-distance movement of the virus (Murota et al. 2017). It is reported to produce several disease symptoms such as leaf mosaic, chlorosis, leaf deformation, necrosis, and stunting. The development of disease symptoms mainly depends upon the host species and the infecting CMV strain (Mochizuki et al. 2014). The functional characterization of the *AO* gene revealed its role in limitation of viral infection, suggesting that it might significantly contribute to resistance against CMV infection.

## Materials and methods

### Plant materials, growth conditions, and virus isolate used in the bioassays

*N. benthamiana* plants were grown in a growth chamber at 25/22 ˚C day/night temperature and 16/8 h light/dark photoperiod. 24-day-old seedlings, typically attaining the four-leaf stage, were selected for TRV application in order to perform VIGS assays for the generation of transiently silenced lines. Subsequent CMV inoculation was performed at approximately 5-week-old plants, typically on the 6th leaf. We measured the plant height of the different groups of plants before and after CMV inoculation, at five sequential time points (once per week). The first two time points were at 1- and 2-weeks post TRV application (thus prior to CMV infection), and the next three time points were at 1-, 2-, and 3-weeks post-CMV inoculation. For each experiment, three biological replications for each treatment were carried out. The overall experimental procedure is presented in Fig. S1.

The CMV isolate used in the experiments, designated as CMV-G and belonging to the CMV subgroup IB, was described previously (Sclavounos et al. 2006). For virus maintenance, *N. tabacum* leaves exhibiting typical CMV-mosaic symptoms (at 14–21 days post-inoculation [dpi]) were ground with water in a mortar at a 1:4 ratio (gr of tissue to ml of water), with the produced leaf sap further diluted to a final 1:50 ratio and inoculated onto carborundum-dusted leaves of newly grown *N. tabacum* seedlings for propagation. The inoculated leaves were washed under running water thrice to remove the traces of carborundum and avoid dehydration of the wounded leaves due to mechanical inoculation via rubbing.

### Phylogenetic analysis of ascorbate oxidase proteins

Protein sequences of putative ascorbate oxidase (AO) proteins from several plant species were retrieved from the Solanaceae Genomics Network [SGN (https://solgenomics.net/)] and the UniProt database (https://www.uniprot.org/). From *N. tabacum* (designated Nt) and *N. benthamiana* (designated Nb) were retrieved the sequences of NtAO_Q40588, NbAO_Niben101Scf03483g00002.1 (Kumari et al. 2016), NtAO_A0A1S3Y315, NtAO_A0A1S3ZI67, NbAO_Niben101Scf03026g01009.1 (this study), and NtAO_A0A1S3YXX8, NtAO_A0A1S3XQJ8. In addition, we retrieved the sequences of AtAO_Q8LPL3, AtAO_O04947, AtAO_O65670 (*A. thaliana*), CsAO4_P14133 (Kumari et al. 2016) (*Cucumis sativus)*, GmAO_A0A0R0EDE6 (*Glycine max)*, OsAO_Q5Z5T3 (Wu et al. 2017), ORAP1_Q69QG3 (Ueda et al. 2015), OsAO_Q5Z645, OsAO_Q0J0J1 (Hu et al. 2022) (*Oryza sativa*), and ZmAO_C0P4R6 (*Zea mays*). The accession numbers of the above-mentioned proteins were obtained from the UniProt database, except proteins from *N. benthamiana* whose accession numbers were obtained from the SGN database (https://solgenomics.net/tools/blast/?db_id=266: Select BLAST database *N. bethamiana* Genome v1.0.1 predicted proteins, paste the designation of the gene e.g. Niben101Scf03026g01009.1 in the Extract sequences from BLAST databases box, search). These sequences were used for phylogenetic analysis; the dendrogram was constructed by the neighbor-joining method with Poisson substitution model, employing the Mega11 software (Tamura et al. 2021). Protein motifs were determined via the InterPro database (https://www.ebi.ac.uk/interpro/) to confirm the presence of conserved motifs in NbAO_Niben101Scf03026g01009.1.

### Design and production of TRV-based engineered constructs for VIGS

The TRV-based VIGS system, developed by Liu et al. (2002), was employed for the production of an engineered VIGS plasmid which could be suitable for the silencing of a putative ascorbate oxidase (*AO*) gene in *Nicotiana* species. TRV is a bipartite plant virus; the pTRV2 binary plasmid vector, which contains the TRV-RNA2 genome, was digested by *Bam*HI (New England Biolabs, Ipswich, MA, USA) to obtain a linearized plasmid, the digestion product was electrophoresed for confirmation and quantification and the digested vector was stored at −20 °C for future use.

For the insertion of an *AO* gene fragment from *N. tabacum*, we employed the In-Fusion seamless cloning system (Takara Bio Inc., Shiga, Japan). Firstly, we used the nucleotide sequence of a putative *AO* gene (coding for protein NtAO_A0A1S3Y315) as a query in the VIGS tool of SGN in order to find the most suitable fragment for gene silencing. From the VIGS tool output, we designed primers for the amplification of a fragment of the *AO* gene (362 nt of CDS in length) using primer3 (https://primer3.ut.ee/). 15-nt extensions, homologous to the linearized pTRV2 vector, were incorporated at the 5’ end of the designed primers to obtain the In-Fusion primers (Table 1). The *AO* gene fragment was amplified by standard RT-PCR reaction, using RNA from *N. tabacum* plants as a template. The PCR product was analyzed by 1.5% agarose gel electrophoresis for confirmation and quantification and then was cloned into the pTRV2 vector following the manufacturer's instructions (Irwin et al. 2012). The In-Fusion reaction mixture product (2 µl) was transformed into *E. coli* Stellar™ competent cells (Takara Bio Inc.), following a heat shock at 42 °C for 60 s. The bacterial cells were plated onto Luria–Bertani (LB) agar plates containing kanamycin (50 µg/ml) and incubated overnight at 37 ^ο^C for colony development and subsequent screening. Colony PCR was performed by using specific primers, TRV2-1530F (forward) and TRV2-1809R (reverse) that bind on the pTRV2 plasmid vector (Table 1). The successful cloning gave a product of 642 nt in length. Two colonies were selected for further multiplication for storage and plasmid isolation by using a plasmid isolation kit (Macherey–Nagel, Düren, Germany). The successful insertion of the *NtAO* fragment into pTRV2 was confirmed by Sanger DNA sequencing (Azenta Life Sciences UK, Essex, UK).

**Table 1:** List of primers used in this study

Name of primer	Sequence (5′ to 3′)	Product size (nt)	Target species	Purpose
Nt_Asc_ox-INF-359F^*^ Nt_Asc_ox-INF-720R^*^	GCCTCCATGGGGATCTTGTTGTTGATAGGCCTGGAA GCTCGGTACCGGATCTCTAAGCCTATAAGTCTTGC	392	*N. tabacum* *N. benthamiana*	Cloning of an *AO* gene fragment to pTRV2
eGFP-INF-286F^*^ eGFP-INF-646R^*^	GCCTCCATGGGGATCGAGCGCACCATCTTCTTCAA GCTCGGTACCGGATCGCTTCTCGTTGGGGTCTTTG	391	–	Cloning of an *eGFP* gene fragment to pTRV2
TRV2-1530F TRV2-1809R	GTTTTTATGTTCAGGCGGTTC TCAAGATCAGTCGAGAATGTCA	280	*E. coli* *A. tumefaciens* agroinfiltrated plant species	Colony PCR, Detection of TRV2
TRV1-257F TRV1-491R	GCTGAGCAGAGGAGTCATTTC ACCCATGAACCATGTTTTTGT	235	*E. coli* *A. tumefaciens* agroinfiltrated plant species	Detection of TRV1
Nt_Asc_ox-728F Nt_Asc_ox-800R	GCTTGACTGCTCTGTCTGCTC TGACCATCTGCCTCAACAACTG	73	*N. tabacum* *N. benthamiana*	Gene expression analysis for *AO*
qF-BOX_N.ben_F qF-BOX_N.ben_R	GGCACTCACAAACGTCTATTTC ACCTGGGAGGCATCCTGCTTAT	127	*N. tabacum* *N. benthamiana*	Gene expression analysis for *F-box*
CMV-CP-F CMV-CP-R	GGGGATCCATGGACAAATCTGAATC GGGGATCCTCAAACTGGGAGCAC	673	CMV	Gene expression analysis for CMV* CP*

^*^The region that anneals to the TRV2 vector for the In-Fusion cloning is underlined

As a negative control in the VIGS bioassays, we cloned a fragment (361-nt in length) of the enhanced GFP (*eGFP*) gene (i.e., a gene that does not exist in plants) into the pTRV2 plasmid vector. To obtain the insert fragment, PCR amplification employing specifically designed In-Fusion primers was done using a pBIN61-eGFP plasmid vector as a template. All other procedures for the production of an engineered TRV::eGFP plasmid were done as described above.

In order to have a visual marker for the efficiency of the VIGS protocol, we used an engineered TRV2 plasmid (courtesy of Prof. Supriya Chakraborty, JNU, New Delhi, India) harboring a fragment of the *N. benthamiana* phytoene desaturase (*PDS*) gene. PDS is an essential factor in the biosynthetic pathway of carotenoids, thus its silencing creates a photobleaching appearance of the leaves, this being used as an indicator for a successfully accomplished experimental procedure.

### Transformation and screening of Agrobacterium harbouring VIGS constructs

*Agrobacterium tumefaciens* competent cells (strain GV3101) were transformed by adding 0.5 μg of purified pTRV2-engineered plasmids. The transformation was carried out following the freeze–thaw transformation protocol, with minor modifications (Weigel and Glazebrook 2006). Briefly, the plasmids were placed into the surface of frozen GV3101 competent cells and immediately placed on a heat block at 37 °C for 5 min. While in the heat block, a brief mixing by pipetting was done once. After heat treatment, the transformed GV3101 competent cells were mixed with LB medium, kept for shaking at 28 °C for 4 h, plated on antibiotic containing kanamycin (50 µg/ml) LB and incubated for 4 days at 28 ^ο^C for colony development. Confirmation for the successful transformation was done by colony PCR as described above for *E. coli*. In a similar manner, the pTRV1 binary plasmid vector (harboring the TRV-RNA1 genome) was also transformed to GV3101 competent cells.

### Virus-induced gene silencing of *N. benthamiana* plants

Virus-induced gene silencing was performed by the agroinfiltration of 24-day-old *N. benthamiana* plants with transformed Agrobacterium cells carrying the produced pTRV2-based constructs along with Agrobacteria carrying the pTRV1 plasmid. Primary and secondary cultures of the respective Agrobacteria were grown in LB medium supplemented with kanamycin (50 µg/ml) at 28 ^ο^C with constant overnight shaking at 200 rpm. The secondary cultures were centrifuged and the supernatant was discarded. The precipitated cells were resuspended in resuspension buffer (10 mM MES-KOH, pH 5.6, 10 mM MgCl_2_). The optical density was measured at 600 nm. Preliminary experiments using different concentrations of Agrobacteria cells were performed, in order to determine the most suitable conditions for agroinfiltration. Agroinfiltration was done on the half of the lamina of one, fully expanded leaf per plant, by pressing against the abaxial leaf surface with a needleless syringe. Plants were kept in the growth chamber for the following 5 weeks, for RNA analysis, CMV inoculation, and symptom observation.

In a typical VIGS bioassay of this study, plants were divided into four groups according to which mixture was infiltrated to them: a) GV3101:TRV1 + GV3101:TRV2::NtAO, b) GV3101:TRV1 + GV3101:TRV2::eGFP, c) Mock, d) GV3101:TRV1 + GV3101:TRV2::NbPDS. Mock-treated plants were treated with resuspension buffer, while group d was employed for the detection of photobleaching in leaves, as an indicator for the efficiency of the VIGS protocol. For simplicity reasons, groups a, b, and d, are mentioned throughout the manuscript as TRV::NtAO, TRV::eGFP, and TRV::NbPDS, respectively.

### Ascorbate oxidase enzymatic assay

The measurement of ascorbate oxidase enzyme activity was performed following the protocol described by Wu et al. (2021). Briefly, systemic leaf tissue (0.5 g) from the TRV::NtAO-, TRV::eGFP- and mock-treated plants was homogenized in 5 ml of 100 mM sodium phosphate buffer (pH 6.5), and after centrifugation at 15,000 g the supernatant was kept on ice for measurement of the soluble AO activity. The pellet was resuspended in 100 mM sodium phosphate buffer (pH 6.5), 1 M NaCl with vortexing, and after centrifugation at 15,000 g the supernatant was kept for measurement of the ionically bound AO activity. The AO assay was done by mixing 80 μl AO assay buffer (100 mM sodium phosphate buffer, pH 5.6), 10 μl plant extract (from the soluble or the ionically bound fraction) and 10 μl of 2 mM ascorbic acid (AA) (AppliChem GmbH, Darmstadt, Germany). The reduction in the absorbance of AA was measured at 265 nm. Since the product of ascorbate oxidase reaction, dehydroascorbic acid (DHA) does not absorb at 265 nm, the reduction at 265 nm is proportional to the oxidation of AA. The determination of protein content in the plant extracts was done using the Bradford method (Kruger 1994). The AO enzymatic activity was calculated as nmol of oxidized AA min^−1^ mg^−1^ of protein in the plant extracts. Detectable AO activity was found only in the ionically bound fraction of plant extracts.

### Gene expression analysis

Total RNA isolation was performed using the TRI Reagent® (Molecular Research Center Inc., Cincinnati, OH, USA) as described previously (Kaldis et al. 2018). For leaf tissue sampling, parts of systemic leaves from 3 plants were joined to acquire bulk samples. Tissue sampling for the investigation of *NbAO* silencing was done at 12 days post application (dpa) of TRV. Tissue sampling for the detection of CMV *CP* (coat protein) was done at 3 days post-CMV inoculation (dpi). RNA concentration of each sample was determined spectrophotometrically by a Multiskan FC Photometer (Thermo Fisher Scientific, Waltham, MA, USA). The quality and integrity of the isolated total RNA were checked by agarose gel electrophoresis. cDNA was synthesized using 200 ng total RNA in a 10 μl reaction volume, employing the FIREScript cDNA synthesis kit (Solis BioDyne, Tartu, Estonia) with oligo-dT and random primers. The RT reaction was performed in a thermal cycler (NIPPON Genetics EUROPE, Düren, Germany) at 27 °C for 10 min, 37 °C for 60 min, and 85 °C for 5 min. PCR amplification for the detection of *NbAO*, *NbF-box*, CMV *CP*, TRV1, and TRV2, was done employing FirePol DNA Polymerase (Solis BioDyne) and specific primers (Table 1). For *NbAO*, we used two primer pairs (Nt_Asc_ox-728F with Nt_Asc_ox-800R; Nt_Asc_ox-INF-359F with Nt_Asc_ox-800R) both giving similar results. The PCR conditions were: 95 ^ο^C for 2 min (initial denaturation), followed by cycling at 95 ^ο^C for 20 s, 60 ^ο^C for 20 s, and 72 ^ο^C for 1 min; the final extension step was at 72 ^ο^C for 8 min. For semi-quantitative gene expression analysis, the PCR-amplified products were analyzed via agarose gel electrophoresis. Quantitative PCR (qPCR) analysis was performed in a StepOnePlus™ Real-Time PCR System (Thermo Fisher Scientific), employing the 5Χ HOT FIREPol EvaGreen qPCR Supermix (Solis BioDyne). Relative quantification of gene expression was carried out using the 2^−∆∆Ct^ method according to Schmittgen and Livak (2008). The Ct values for CMV *CP* in the mock-treated plants were undetermined, and were arbitrary set as 40 PCR cycles. The statistical analysis was done using the Student’s *t*-test. The statistically significant difference of relative expression levels among the different groups of plants was determined at *P* < 0.05.

## Results

### Selection of a new ascorbate oxidase gene in *N. benthamiana*

The hypothesis that *AO* genes could be generally involved in plant viral infections was tested employing our pathosystem of choice, namely the CMV-*N. benthamiana*. This is because we could use a well-described CMV strain studied in our lab (Sclavounos et al. 2006) and because *N. benthamiana* is a very suitable plant species for performing VIGS for functional analysis (Liu et al. 2002). *AO* genes in plants belong to a multigene family. We selected for silencing a *NbAO* gene (Niben101Scf03026g01009.1), which shares 91–92% identity at the gene and protein level with the two ortholog genes in *N. tabacum* (producing the NtAO_A0A1S3Y315 and NtAO_A0A1S3ZI67) that were found to be induced early in the TMV infection process in tobacco (Voloudakis’ lab unpublished data). Niben101Scf03026g01009.1 is substantially different from NbAO_Niben101Scf03483g00002.1 (studied by Kumari et al. 2016), exhibiting only 58% and 46% identity at the gene and protein level, respectively.

### Induction of *NbAO* expression in response to CMV infection

**Fig. 1:**
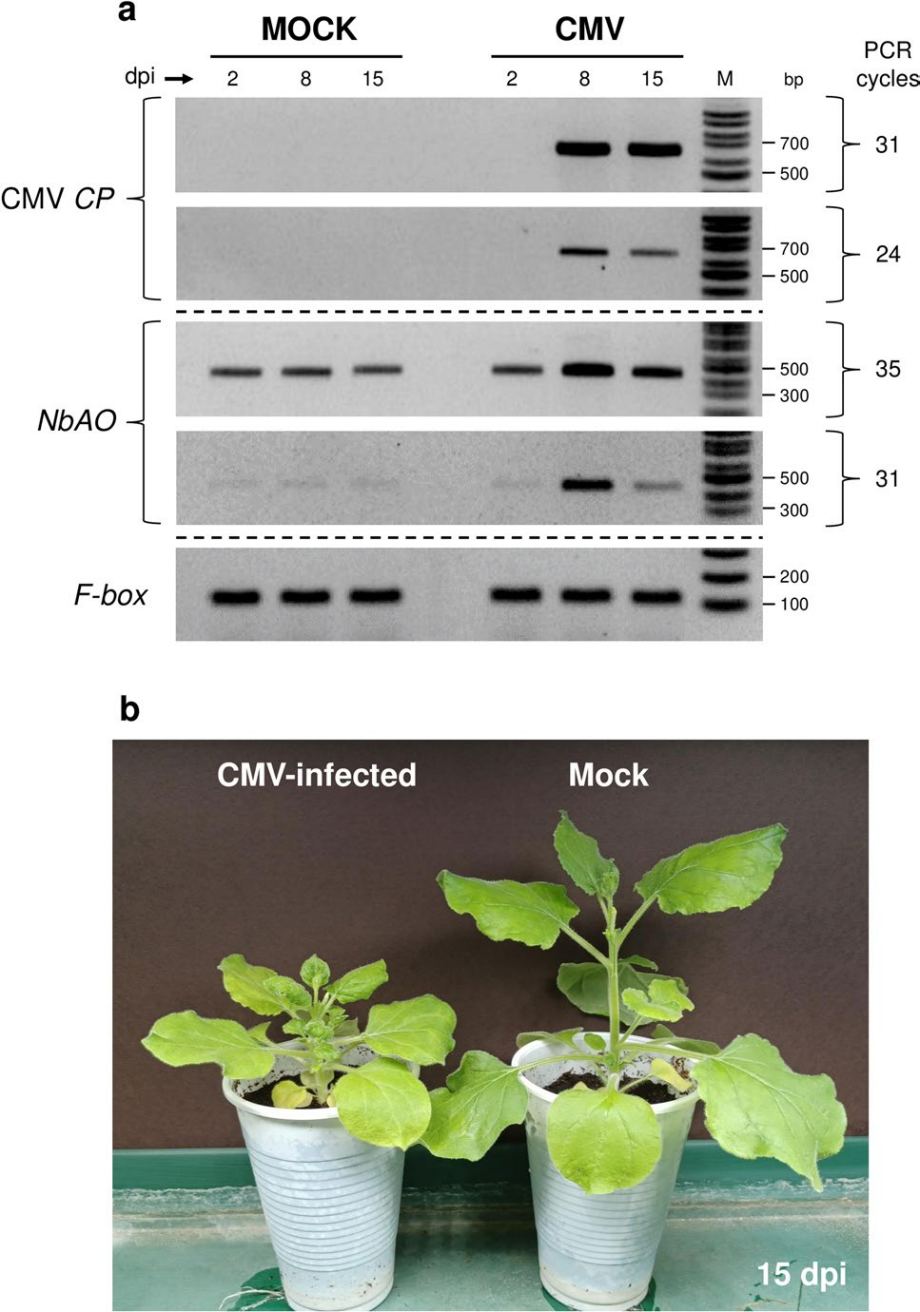
CMV infection correlates with increased expression of an ascorbate oxidase gene in systemic leaves of *N. benthamiana* plants. **a** Semi-quantitative RT-PCR analysis for the detection of CMV *CP* and NbAO_Niben101Scf03026g01009.1 levels at mock- and CMV-inoculated plants at 2, 8, and 15 dpi. The experiment was repeated thrice and representative gel blots are shown. The detection of the *F-box* housekeeping gene was used as an internal control. For CMV *CP* and *NbAO*, results from PCR amplification at two different PCR cycles are shown. Lane M represents a 100 bp molecular ladder (NIPPON Genetics EUROPE, Düren, Germany). **b** Stunted growth of CMV-infected *N. benthamiana* plants at 15 dpi, as compared to the mock-treated control plants

To explore whether CMV infection affects the expression levels of the NbAO_Niben101Scf03026g01009.1 gene, 5-week-old *N. benthamiana* plants were divided in two groups, with the first group being inoculated with CMV and the second where H_2_O was applied (mock). Semi-quantitative gene expression analysis was carried out at three time points, i.e., at 2, 8, and 15 dpi, in the systemic (non-treated) leaves of the inoculated plants. CMV titer was determined by RT-PCR amplification of the CMV *CP* gene (Fig. 1a). CMV *CP* was undetectable at 2 dpi, suggesting that no significant replication and systemic movement of CMV had occurred by this early time point. In contrast, a very strong band was detected at 8 and 15 dpi, indicating a dramatic increase in CMV titer. The accumulation peak of CMV was at 8 dpi, while a small decline occurred at 15 dpi. At the time points of high CMV titer, the development of CMV disease symptoms were obvious, as evidenced by the stunted growth of CMV-inoculated plants in comparison to the healthy control plants (Fig. 1b).

PCR amplification with *NbAO*-specific primers produced a distinct band in both mock- and CMV-inoculated plants (Fig. 1a). In the mock-treated plants, the *NbAO* levels were rather low and no significant variation was observed among the three time points, suggesting that the expression of *NbAO* in leaves remains stable in the time period of the experimentation. However, significantly higher levels of *NbAO* were observed in the CMV-inoculated plants at 8 and 15 dpi, but not at 2 dpi. The peak of *NbAO* induction was at 8 dpi, suggesting that the *NbAO* expression levels increase in parallel with the CMV titer (Fig. 1a) indicating a correlation of CMV infection with the induction of *NbAO* in *N. benthamiana*. CMV is inducing a transcriptional reprogramming of the host plant (Chen et al. 2017). The arising question is whether the *NbAO* induction is part of a defense response of the host plant to CMV?

### Phylogenetic analysis of NbAO

**Fig. 2:**
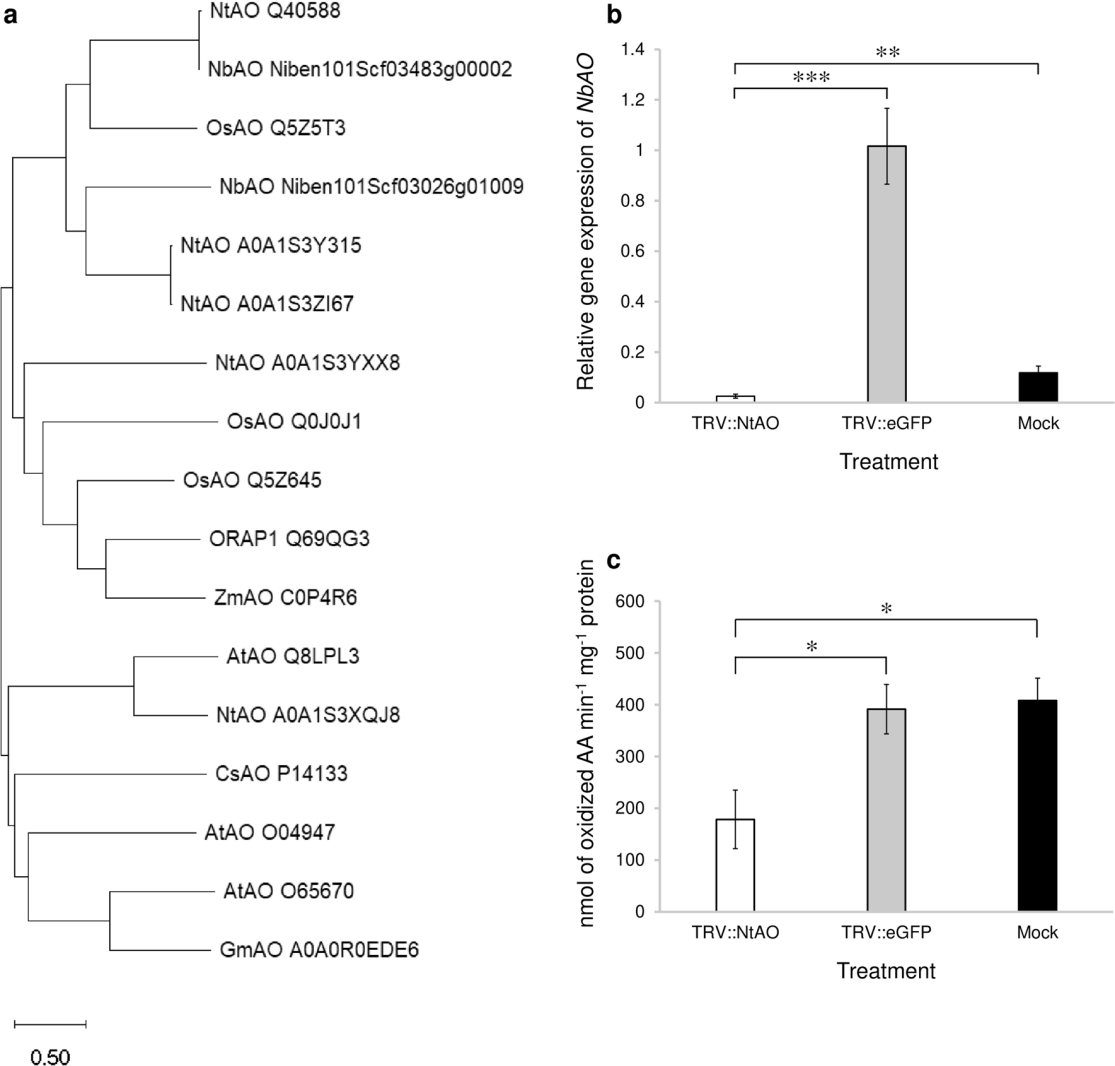
Ascorbate oxidase (*AO*) gene and function analysis. **a** Phylogenetic analysis of ascorbate oxidase-(AO) related proteins from several plant species, employing the neighbor-joining method. The branch length is proportional to the evolutionary distance among the different AO proteins. **b** Silencing of the NbAO_Niben101Scf03026g01009.1 gene in *N. benthamiana* employing a TRV-based VIGS approach. qPCR analysis was done for the quantification of the *NbAO* expression levels. RNA isolation was carried out at 12 days post TRV or mock application (see “Materials and methods”). **c** Measurement of AO enzymatic activity in the TRV::NtAO-treated plants as compared to the control groups (see “Materials and methods”). For **b** and **c**, three biological replicates were performed and the results in the graphs are depicted as the mean ± standard error (*n* = 3). White column: TRV::NtAO; grey column: TRV::eGFP; black column: Mock. Statistical analysis was performed using the Student’s *t*-test. Asterisks indicate that the mean values between different groups differ significantly (* *P* < 0.05, ** *P* < 0.005, *** *P* < 0.001)

To determine the phylogenetic relationship of NbAO with other AO-related proteins from several plant species (see M&M), we performed a phylogenetic analysis (Fig. 2a). NbAO_Niben101Scf03026g01009.1 was found to be in the same clade as its tobacco orthologs NtAO_A0A1S3Y315 and NtAO_A0A1S3ZI67. These three proteins were found to be in close relationship with the NbAO_Niben101Scf03483g00002.1 and NtAO_Q40588 (studied by Kumari et al. 2016), as well as with OsAO_Q5Z5T3 which is the most typical ascorbate oxidase in rice (Wu et al. 2017). In contrast, other putative AO proteins from *N. tabacum* were found to be in clearly diversified clades. For example, NtAO_A0A1S3YXX8 was found to be related to several AO-related proteins from monocot species, like ORAP1_Q69QG3 (studied by Ueda et al. 2015).

The analysis of protein motifs for NbAO_Niben101Scf03026g01009.1, done via the InterPro, indicated that typical motifs of AOs, such as the N-terminal domain (identifier IPR011707), the 2nd and 3rd cupredoxin domains (identifiers IPR001117 and IPR034267, respectively) and the copper-binding site (identifier IPR002355) were present (Fig. S2).

### Optimization of VIGS protocol and effective silencing of *NbAO* gene using VIGS

For the functional characterization of the *AO* gene of *N. benthamiana* (*NbAO*), VIGS was performed employing a TRV-based construct carrying a fragment of the *NtAO* gene. The efficiency of the TRV-based VIGS protocol was maximized by employing the visualization of photobleaching appearance of leaves upon VIGS of *NbPDS* in *N. benthamiana* (Fig. S3). Agrobacteria having O.D_600_ = 0.6 consisted the most efficient concentration, as evidenced by the almost complete whitening of the leaves (Fig. S3a) and was used in all agroinfiltrations. Regarding the timing of the induced silencing, the preliminary assays indicated that the onset of photobleaching at the newly emerging leaves occurred at 7 dpi, while sufficient downregulation of the *NbPDS* gene was accomplished as early as 12 dpi (Fig. S3b).

To detect the downregulation of *NbAO*, both semi-quantitative and quantitative RT-PCR were performed at 12 days post TRV application (dpa). The semi-quantitative RT-PCR revealed efficient silencing of *NbAO* gene (Fig. S4), producing a very faint band in the TRV::NtAO-treated plants in comparison to the control plants (TRV::eGFP- and mock-treated). The rather low expression levels of *NbAO* in mock-treated plants were in agreement with the results from Fig. 1, indicating low basal expression levels. On the contrary, *NbAO* expression seemed to be highly induced by the TRV::eGFP treatment. Further, quantification of the *NbAO* expression levels using RT-qPCR (Fig. 2b) confirmed the significant downregulation in the TRV::NtAO virus-induced silenced plants in comparison to the control groups (TRV::eGFP- and mock-treated plants), indicating that the VIGS protocol targeting the *NbAO* gene was as reliable as the one targeting the *NbPDS* gene (Fig. S3). The relative quantification values for TRV::AO-, TRV::eGFP-, and mock-treated groups were found to be 0.025, 1.016, and 0.118, respectively (Fig. 2b). This corresponds to a 97.5% downregulation of the *NbAO* gene in the TRV::NtAO-treated plants with respect to the TRV::eGFP-treated plants (statistically significant at *P* < 0.001). The downregulation relative to the mock-treated plants was 78.8% (statistically significant at *P* < 0.005).

### Reduction of ascorbate oxidase enzyme activity in the *AO*-silenced *N. benthamiana* plants

To determine the levels of ascorbate oxidase enzymatic activity under the silenced condition i.e., in the TRV::NtAO-treated plants in comparison to the control groups (TRV::eGFP and mock), the AO enzyme assay was performed. The levels of ascorbate oxidase enzymatic activity (calculated on the basis of the rate of AA oxidation) was found to be 178.4, 391.3, and 408 nmol of oxidized AA min^−1^ mg^−1^ of protein in the plant extracts of TRV::NtAO-, TRV::eGFP-, and mock-treated plants, respectively, indicating lower levels of AO enzymatic activity in the *NbAO*-silenced plants in comparison to the control groups (Fig. 2c), suggesting that the downregulation of NbAO_Niben101Scf03026g01009.1 gene resulted in less accumulation of AO enzyme in *N. benthamiana*. The difference between the TRV::NtAO-treated plants and the two control groups was found to be statistically significant at *P* < 0.05.

### Effect of the *AO*-silencing in *N. benthamiana* to CMV disease phenotype

**Fig. 3:**
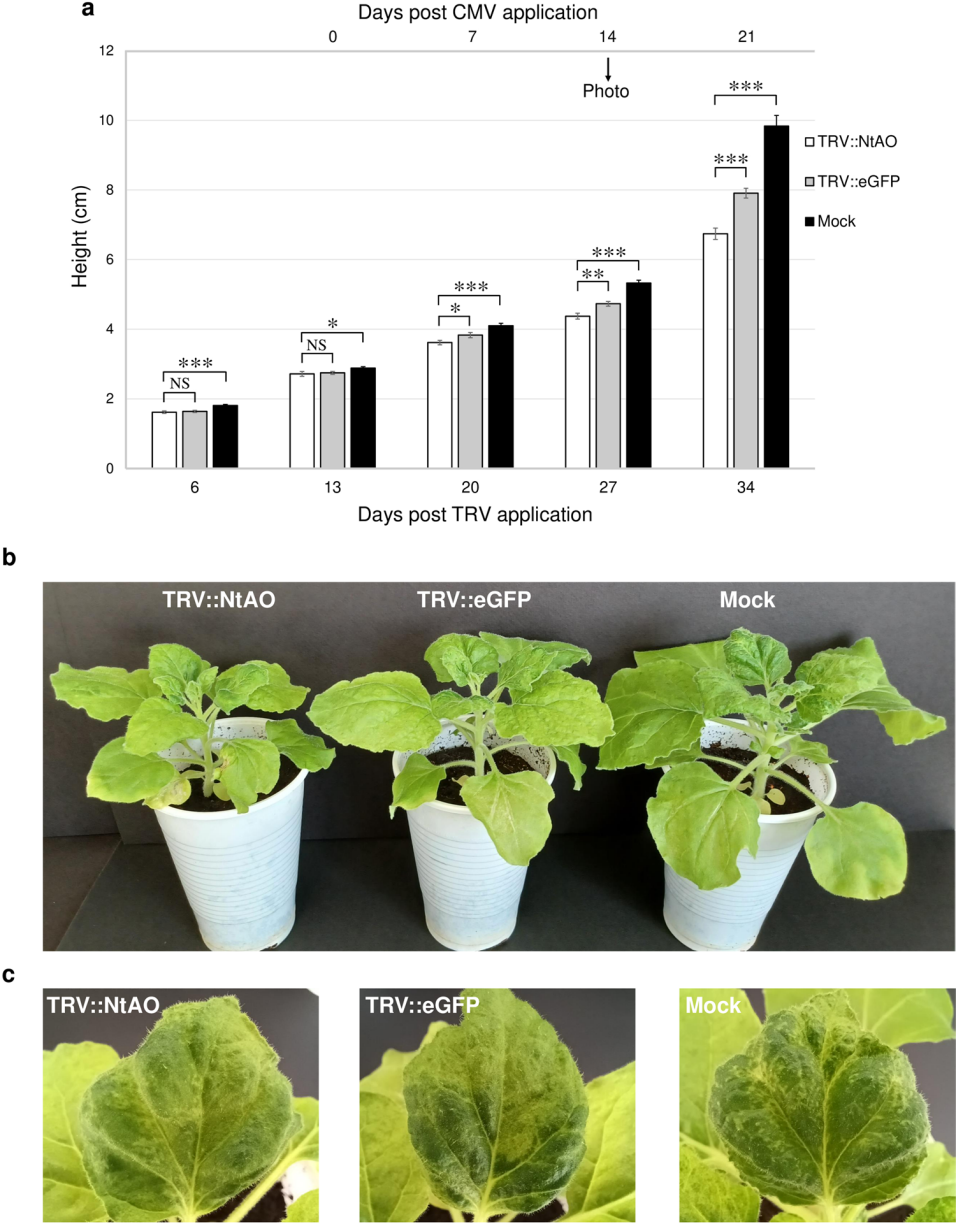
Effect of *AO*-silencing in CMV disease development in *N. benthamiana* plants. **a** Reduced height of the *NbAO*-silenced lines as a consequence of CMV inoculation. The time points are shown at the lower X axis. CMV inoculation was performed at 13 days post TRV application. At 1, 2 and 3 weeks post-CMV inoculation, a significant difference in the height of TRV::NtAO-treated (as compared to the TRV::eGFP-treated) plants is evidenced. The time points after CMV inoculation are shown at the upper X axis. Columns in the histogram represent the mean ± standard error (*n* = 12). White column: TRV::NtAO; grey column: TRV::eGFP; black column: Mock. Statistical analysis was performed using the Student’s *t*-test. Asterisks indicate that the mean values between compared groups differed significantly (* *P* < 0.05, ** *P* < 0.005, *** *P* < 0.001). NS indicates that there was no statistically significant difference. The black arrow indicates the time point the photos (in panels **b** and **c**) were taken. **b** Appearance of TRV::NtAO-, TRV::eGFP-, and mock-treated plants at 14 days post-CMV inoculation. Dwarfism is evident as a hallmark of CMV disease development. **c** Appearance of individual leaves of CMV-inoculated *N. benthamiana* plants, exhibiting mosaic and malformations. Representative images from leaves of the TRV::NbAO-, the TRV::eGFP-, and the mock-treated plants, are shown at 14 days post-CMV inoculation

In order to investigate the involvement of AO in *N. benthamiana*-CMV interaction, we assessed the CMV disease development in the *NbAO*-silenced lines. As described in Fig. 2b, a significant silencing of the *NbAO* gene was achieved at 12 days post-TRV application. No significant difference in height was evidenced between the TRV::NtAO- and the TRV::eGFP-treated *N. benthamiana* plants (Fig. S5). The effect of TRV infection (TRV::NtAO- and TRV::eGFP-treated plants) to *N. bethamiana* was estimated to be 23% reduction in plant height (34 dpa) (Fig. S5). Thus, TRV although being a generally mild virus, reduces the growth of *N. benthamiana* plants. We challenged plants with CMV at 13 days post-TRV application. The natural growth of the plants inoculated with CMV was reduced when *NbAO* was silenced (in the TRV::NtAO-treated plants) as compared to TRV::eGFP-treated plants. In particular, the mean values of plant height for the TRV::NtAO-, the TRV::eGFP-, and the mock-treated plants at 34 dpa (post TRV) or 21 dpi (post-CMV) were 6.7, 7.9, and 9.8 cm, respectively (Fig. 3a, Fig. 3b). In other words, silencing of *NbAO* gene resulted in the appearance of a more stunted plant growth by 15.2% upon CMV infection. At the same time, CMV infection also causes distinct mosaic and malformations in leaves of TRV::NtAO-, TRV::eGFP-, and mock-treated plants (Fig. 3c), but no obvious difference in the severity of the CMV disease symptoms among the three groups is observed at 14 dpi (Fig. 3c).

### Determination of CMV and TRV titers in the CMV-challenged *N. bethamiana* plants

**Fig. 4:**
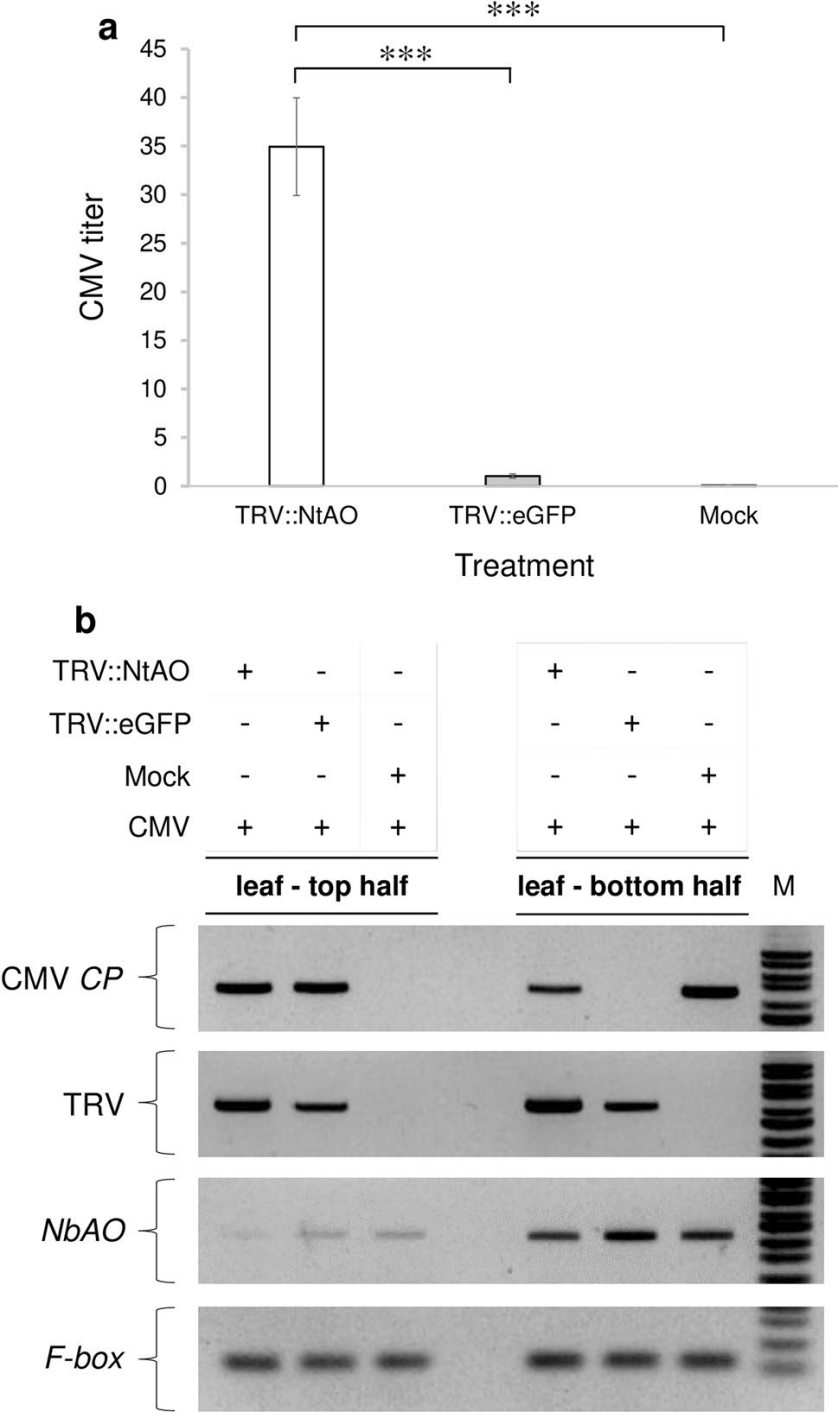
Expression levels of CMV, TRV, and *NbAO* in the systemically CMV-infected leaves in *N. benthamiana* plants. **a** Quantification of CMV titer by qPCR analysis in the systemic leaves of the treated plants. CMV inoculation was performed on the 6th leaf of all plants. RNA isolation was carried out at 3 days post-CMV inoculation from the 9th leaf of plants (see Fig. S1). The quantification was done following the 2^−∆∆Ct^ method, using F-box for normalization purposes (see “Materials and methods”). The columns represent the mean value ± standard error (*n* = 3). White column: TRV::NtAO; grey column: TRV::eGFP; black column: Mock. The Ct values for CMV *CP* in the mock-treated plants were undetermined and thus were set arbitrary to 40 PCR cycles. Statistical analysis was performed using the Student’s *t*-test. Asterisks indicate that the mean values between different treatments differ significantly (*** *P* < 0.001). **b** Variability in the CMV and TRV levels at the systemic leaves of TRV::NtAO-treated plants as compared to the control groups (TRV::eGFP- and mock-treated plants) depending on the part of leaf lamina. CMV application and RNA isolation was carried out as mentioned in **a**, with the exception that sampling was done from the 8^th^ leaf of plants (see Fig. S1). Moreover, this leaf being big enough, was divided in two parts: the top and the bottom half, for RNA isolation. Gel blots for the detection of CMV *CP*, TRV, *NbAO*, and *F-box* are presented. Above the gel blots, are shown the applied treatments that correspond to each column. For details see “Materials and methods”. Lane M represents a 100 bp molecular ladder (NIPPON Genetics EUROPE, Düren, Germany)

To determine the CMV titer in the *NbAO*-silenced, both semi-quantitative and real-time RT-PCR analysis using CMV-specific primers (Table 1) were done. CMV inoculation was carried out on the 6th leaf and tissue sampling was done from the 9th leaf in order to monitor the magnitude of the systemic spread of CMV (Fig. S1). The semi-quantitative gene expression analysis revealed significantly greater accumulation of CMV in the *NbAO-*silenced group (TRV::NtAO) in comparison to the TRV::eGFP and mock (Fig. S6) at 3 dpi. This was validated by the relative quantification of CMV *CP* gene that showed a high upregulation (34.9-fold increase) in the TRV::NtAO-treated plants in comparison with the TRV::eGFP group, with the latter set as having relative quantity of 1 (Fig. 4a). The results were found to be significantly different even at *P* < 0.001, pointing out that the silencing of the NbAO_Niben101Scf03026g01009.1 gene renders the *N. benthamiana* plants more susceptible to CMV multiplication, contributing to the extra stunted growth mentioned above.

Our experimental design (Fig. S1) of the two sequential viral inoculations, firstly with TRV and secondly with CMV triggered several intriguing questions. Does TRV act in conjunction with CMV to facilitate the spreading of the latter? Alternatively, is there any antagonism between TRV and CMV for effective accumulation in different parts of the plants? To answer these, we performed sampling from different portions of the same leaf to check whether there was any variability in the accumulation of CMV and TRV. RNA was extracted from the 8th leaf of plants (Fig. S1) at 3 days post-CMV inoculation. Being large in size, the 8th leaf was divided in two parts (the top and the bottom half). The detection of CMV *CP* and TRV in the two parts of the leaf was done using specific primers (Table 1) by performing semi-quantitative RT-PCR. Surprisingly, the results showed a great variability in CMV and TRV titers depending on the part of the leaf sampled and the specific treatments applied to the plants (Fig. 4b). Firstly, the mock-treated plants exhibited a high CMV accumulation only at the bottom half of the leaf, whereas CMV was absent from the top half of the leaf. These results indicated that CMV spreading in systemic leaves in WT *N. benthamiana* plants is not uniform to the whole leaf lamina. Interestingly, in the TRV::eGFP-treated plants, the CMV accumulation pattern was found to be exactly opposite to the mock-treated plants; in other words, CMV accumulated only at the top half while at the bottom half of the leaf it was undetectable, indicating that the two control groups (mock and TRV::eGFP) are not biologically equal and significantly differ between them. The most plausible explanation is that in the TRV::eGFP-treated plants, TRV being the first inoculated virus onto the plants moves and preferentially accumulates at the bottom half of the leaf (Fig. 4b), then inhibits the coexistence with CMV, thus CMV accumulating at the top half of the leaf. On the other hand, in the TRV::NtAO-treated plants (*NbAO*-silenced plants), we observed that CMV accumulated to both the top and the bottom half of the leaf, suggesting that the silencing of the NbAO_Niben101Scf03026g01009.1 gene results in the expansion of the spreading area of CMV, supporting the above-mentioned observations of more stunted growth and increase in CMV susceptibility in the transiently *NbAO*-silenced plants. Interestingly, TRV titer was found to be significantly higher in the TRV::NtAO- as compared to the TRV::eGFP-treated plants, either at the top or the bottom half of the leaf, suggesting that the silencing of the NbAO_Niben101Scf03026g01009.1 gene renders the *N. benthamiana* plants more susceptible not only to CMV, but also to TRV. Lastly, the detection of *NbAO* levels verified that the silencing of NbAO_Niben101Scf03026g01009.1 gene still existed even after the challenging with CMV, either at the top or the bottom half of the leaf (Fig. 4b).

## Discussion

### *AO* induction by CMV infection

Ascorbate oxidases (AOs) play major role in plant growth and development including responses to environmental stress and also maintenance of cellular redox homeostasis (Yamamoto et al. 2005; Pignocchi et al. 2006; Garchery et al. 2023; Karpinska et al. 2018; Pan et al. 2019). In the present study, an *AO* gene (NbAO_Niben101Scf03026g01009.1) was induced in the systemic leaves of *N. benthamiana* plants after CMV infection (Fig. 1). This agrees with the general characteristic of defense-related genes that are constitutively produced at basal levels, to avoid fitness cost, and are induced only after pathogen attack. The host specific responses to viruses could lead to the exploration of novel plant targets in order to understand their process of infection that can be subsequently utilized to produce resistant varieties. Likewise, the induction of *CsAO4* expression was reported during CMV infection in *C. sativus* at 36–96 h post-inoculation (hpi) at the inoculated leaves (Kumari et al. 2016). Moreover, the role of AO proteins in plant defense have been reported (Singh et al. 2021). In rice, an *AO* gene plays an important role in the defense against RSV and RBSDV (Wu et al. 2017). Α concurrent accumulation of CMV *CP* and *AO* was observed in the current study in the infected plants. It seemed that *NbAO* could be generally induced upon viral infections since it was also induced by TRV (Fig. 2b). Furthermore, the potential role of AO has been studied in plant nematodes interaction; AO was reported to function as an efficient systemic defense priming agent against nematode infection in sugar beet and rice via induction of multiple basal plant defense pathways (Singh et al. 2020a, b). Further, AO foliar application in rice activated the plant systemic defense response in the rice root without having any negative effect on plant growth (Singh et al. 2021). In the present study, the induction of *AO* gene after CMV infection, might suggest its role in induction of systemic defense response in *N. benthamiana* in response to CMV infection.

### Functional characterization of an *AO* gene in *N. benthamiana* employing VIGS

To validate the functional role of NbAO_Niben101Scf03026g01009.1 in *N. benthamiana*, TRV-based VIGS analysis was performed. Based on the TRV::NbPDS experiments we estimated that a period of 12 days are needed for efficient silencing in *N. benthamiana* with the TRV-based VIGS approach (Fig. S3). The successful silencing of NbAO_Niben101Scf03026g01009.1 was confirmed by both semi-quantitative (Fig. S4) and RT-qPCR analysis (Fig. 2b). We observed a 97.5% to 78.8% reduction in *NbAO* mRNA levels in the TRV::NtAO-treated plants as compared to the control groups (TRV::eGFP and mock) (Fig. S4, Fig. 2b), proving the efficacy of the TRV-based VIGS method in *N. benthamiana*, in agreement to the 90% reduction in transcript level and the appearance of photobleaching at nearly 10 dpa (Senthil-Kumar and Mysore 2014). Furthermore, after agroinoculation, no leaf deformation has been observed in the TRV-treated groups as compared to mock, agreeing with the notion that TRV infection is rather mild in several host plant species (Senthil-Kumar and Mysore 2014).

Furthermore, the lower levels of AO enzymatic activity in the TRV::NtAO group of plants in comparison to the control (TRV::eGFP) suggests an effective silencing of the respective *AO* gene and subsequent reduction of enzyme concentration (Fig. 2c).

*NbAO* silencing does not seem to affect *N. benthamiana* growth since no significant difference in plant height was observed in the TRV::NtAO plants in comparison to the TRV::eGFP plants (Fig. S5). However, after CMV infection, a significant difference in plant height was observed (Fig. 3) in the *NbAO*-silenced plants. It is known that viruses promote stunting of the plants (Islam et al. 2019). The reduced height of TRV::NtAO plants in comparison to the TRV::eGFP plants after CMV inoculation strongly suggest that the *NbAO*-silenced plants became more susceptible to CMV.

### *AO* silencing by VIGS increased CMV accumulation at an early stage of infection in *N. benthamiana*

The CMV titer in the systemic leaves of *N. benthamiana* was higher in *NbAO*-silenced plants, with very low levels of CMV *CP* accumulation in the TRV::eGFP control and relatively no expression in the mock-treated plants (Fig. 4a), an observation that strongly suggest that NbAO_Niben101Scf03026g01009.1 gene functions as a resistant gene against CMV infection. Furthermore, the variation of CMV *CP* levels in top and bottom half of the systemic leaf showed that that there is an antagonism between the two viruses, TRV and CMV. This finding has implications in research efforts where VIGS is employed followed by another virus infection. Ιn TRV::eGFP control plants, CMV accumulated only at the top half, while at the bottom half, CMV was undetectable, mainly due to the fact that this area is occupied by TRV. In contrast, in *NbAO*-silenced plants, CMV accumulated also at the bottom half. The higher accumulation of *CP* gene and the expansion of the spreading area of the leaf lamina in the *NbAO*-silenced group might be due to the easier movement of CMV across the cells resulting in higher CMV titer. Another possibility could be the entrance of CMV in the TRV-infected bottom tissue of the leaf due to the suppression of RNAi in the *NbAO*-silenced group. Our data suggest that the *NbAO* gene has a role in suppressing or limiting CMV infection, acting therefore as a resistant factor against CMV. Wu et al. (2017) proved that AO enzymes, by enhancing Reactive Oxygen Species (ROS) accumulation, promote ROS signaling, i.e., permit ROS to act as secondary messengers in signal transduction pathways resulting in resistance against viral pathogens. Such a study could be done in the *NbAO*-silenced plants produced in the present study.

It should be noted that silencing of another *AO* gene (NbAO_Niben101Scf03483g00002.1) in *N. benthamiana* (Kumari et al. 2016) resulted in a moderate reduction in CMV accumulation in systemic leaves at 5 dpi. There are several possible explanations for this apparent discrepancy between the two studies in *N. benthamiana*. Firstly, since *AO* genes belong to a multigene family, it is very possible to have functional redundancy among different members, thus, the effect of the silencing of a single *AO* gene may be hidden from the action of the remaining members. Secondly, the two studies used CMV isolates belonging to different CMV subgroups: i.e., subgroup I (our study) and subgroup II (Kumari et al. 2016). Differences in the aggressiveness of CMV isolate might have affected the susceptibility of the silenced plants. Lastly, as shown in our study, the viral titer in different areas of the leaf lamina may significantly differ, thus the collection of part of the leaf for RNA extraction may render the CMV titer estimation misleading.

AO proteins exist in the apoplast as homodimers. Kumari et al. (2016) performed bimolecular fluorescence complementation (BiFC) assays showing the localization of movement protein of CMV with AO around the cell wall periphery. They proposed a model in which the association of functional AO dimers with the movement protein of CMV is likely to reduce the dimer formation, thus inhibiting the defense response of the plant. An analogous model was proposed by Hu et al. (2022) regarding the compatible interaction of rice with the blast fungus *Magnaporthe oryzae*: the AO-related protein MoAo1 is secreted by the fungus into the rice apoplast, compromise the functionality of rice AO by the disruption of homodimers, resulting in an imbalance in the redox state of the host’s apoplast that promotes fungal infection. Further work is needed to clarify the specific mechanisms and the signal transduction pathways arising from AO function that leads to the induction of plant defense responses. The identification of AO-interacting proteins or downstream factors could open new directions in the effort to achieve a broad-spectrum resistance of host plants against several plant pathogens.

## Conclusions and future perspectives

This study provided an insight into the defensive role of an *NbAO* gene (NbAO_Niben101Scf03026g01009.1) in response to CMV inoculation. Our results showed that this *AO* gene might be a resistant factor against viral infection since its silencing enhances the spreading of CMV infection and increase plant stunting. The notion that AO proteins are resistant factors could be strengthened by performing simultaneous silencing to more than one *AO* genes. In addition, the putative role of *AO* genes as resistance genes may be further confirmed by their overexpression in *N. benthamiana* plants inoculated with CMV, that could lead to the production of resistant to CMV varieties.

### Supplementary Information

Below is the link to the electronic supplementary material.

Supplementary file1 (DOCX 32 kb)

Supplementary file2 (PDF 903 kb)

## Data Availability

All research data supporting the results and analysis of the article could be shared upon request.

## Abbreviations

AA: Ascorbic acid
AO: Ascorbate oxidase
CMV: Cucumber mosaic virus
PTGS: Post-transcriptional gene silencing
TRV: Tobacco rattle virus
VIGS: Virus-induced gene silencing
