# Supplementary material for: Silencing of a Nicotiana benthamiana ascorbate oxidase gene reveals its involvement in resistance against cucumber mosaic virus
Source: Planta. 2024 Jan 16;259(2):38. doi: 10.1007/s00425-023-04313-x (PMC10791908; doi:10.1007/s00425-023-04313-x)
Supplement: Supplementary file 1 — Supplementary file1 (DOCX 32 kb) [file 425_2023_4313_MOESM1_ESM.docx]

Supplementary Material

**Figure legends**

**Fig. S1**  Schematic graph showing the experimental design for the sequential inoculation of *N. benthamiana plants* with TRV (**a**) and CMV (**b**) followed by RNA isolation at 3 days post CMV (**c**) and height measurement (**d**) to determine the effect of *NbAO* silencing on CMV infection. Plants were inoculated with TRV-based constructs (TRV::NtAO, TRV::eGFP) or with resuspension buffer only (Mock) on the 3^rd^ leaf. After 13 days, a sufficient downregulation of the endogenous *NbAO* gene has been achieved. Thus, this was the ideal time point for CMV inoculation, which was done on the 6^th^ leaf by the application of CMV sap and subsequent rubbing of the leaf via carborundum. At 3 days post CMV inoculation, RNA isolation for the detection of CMV titer was carried out either on the 9^th^ leaf (see Fig. 4a) or on the -much bigger- 8^th^ leaf (see Fig. 4b). Importantly, for tissue sampling the 8^th^ leaf was cut in the middle, and two different RNA isolations were performed, one from the top half and one from the bottom half of the leaf. Height measurement was carried out at three time points after CMV infection (see Fig. 3). Created with BioRender.com

**Fig. S2** Prediction of protein domains in an ascorbate oxidase protein. Amino acid sequence (**a**) and protein motifs (**b**) found along the NbAO_Niben101Scf03026g01009.1 protein. The domains were analyzed via the InterPro database: N-terminal domain found in Multicopper oxidases (IPR011707, a.a 37-96) (orange oval); 2^nd^ cupredoxin domain found in Multicopper oxidases (IPR001117, a.a 134-291) (purple oval); 3^rd^ cupredoxin domain found in Ascorbate oxidases (IPR034267, a.a 336-527) (red oval); copper-binding site found in multicopper oxidases (motif IPR002355, a.a 501-512) (blue box); predicted signal peptide (a.a 1-28) (green oval)

**Fig. S3**  Visualization of TRV-based VIGS efficiency by silencing of the *NbPDS* gene which generates a photobleaching appearance of the leaves. **a** Different concentrations of agrobacteria harboring TRV-based constructs carrying a fragment of the *NbPDS* gene were used for agroinfiltration of *N. benthamiana* plants. The most efficient concentration, as evidenced by the severity of photobleaching of leaves, was accomplished by using agrobacteria having O.D_600_=0.6 (image on the left). A representative, almost white, leaf is highlighted. In contrast, agrobacteria having O.D_600_=1 were not such effective, with the leaves showing a chimeric appearance with green and white regions (image on the right). A representative leaf, showing whitening only at the bottom part along the veins, is highlighted. Photos were taken at 21 days post TRV application (dpa). **b** Photos of *N. benthamiana* plants agroinfiltrated with TRV::NbPDS (O.D_600_=0.6) at 7 and 12 dpa. The orange arrow indicates the onset of photobleaching at the newly emerging leaves at 7 dpa. Photo at 12 dpa indicates that a sufficient downregulation of the *NbPDS* gene has been accomplished

**Fig. S4**  Semi-quantitative RT-PCR analysis showing the silencing of an ascorbate oxidase gene (NbAO_Niben101Scf03026g01009.1) in *N. benthamiana* employing VIGS. The results show significantly lower expression levels in the TRV::NtAO-treated plants in comparison to the control groups (TRV::eGFP- and mock-treated plants). For each treatment, the 3 lines correspond to 3 biological replicates. RNA isolation was carried out from systemic leaves at 12 days post TRV or mock inoculation. The detection of the *F-box* housekeeping gene was used as an internal control. Lane M represents a 100 bp molecular ladder (NIPPON Genetics EUROPE, Düren, Germany)

**Fig. S5**  *NbAO* silencing in *N. benthamiana* plants does not affect the plant height of TRV-infected plants. Plant height was measured for 5 weeks, starting from 6 days post TRV application. The time points are shown at the X axis. Columns in the histogram represent the mean ± standard error (*n* = 5). White column: TRV::NtAO; grey column: TRV::eGFP; black column: Mock. Statistical analysis was performed using the Student’s *t*-test. Asterisks indicate that the mean values between compared groups differed significantly (* *P* < 0.05, ** *P* < 0.005, *** *P* < 0.001). NS indicates that there was no statistically significant difference. No significant difference in height is evidenced between the TRV::NtAO- and the TRV::eGFP-treated plants, while both these groups differ from mock-treated plants

**Fig. S6**  Semi-quantitative RT-PCR analysis of CMV titre in the *NbAO*-silenced plants as compared to the control groups at 3 dpi. The results show significantly higher CMV *CP* expression levels in the TRV::NtAO-treated plants in comparison to the control groups (TRV::eGFP- and mock-treated plants). For each group, the 3 lines correspond to 3 biological replicates. RNA isolation was carried out from systemic leaves at 3 days post CMV inoculation (see Fig. 4a). The gene expression of the F-box housekeeping gene was used as an internal control. Lane M represents a 100 bp molecular ladder (NIPPON Genetics EUROPE, Düren, Germany)
