# Supplementary material for: Silencing of a Nicotiana benthamiana ascorbate oxidase gene reveals its involvement in resistance against cucumber mosaic virus
Source: Planta. 2024 Jan 16;259(2):38. doi: 10.1007/s00425-023-04313-x (PMC10791908; doi:10.1007/s00425-023-04313-x)

Fig. S1

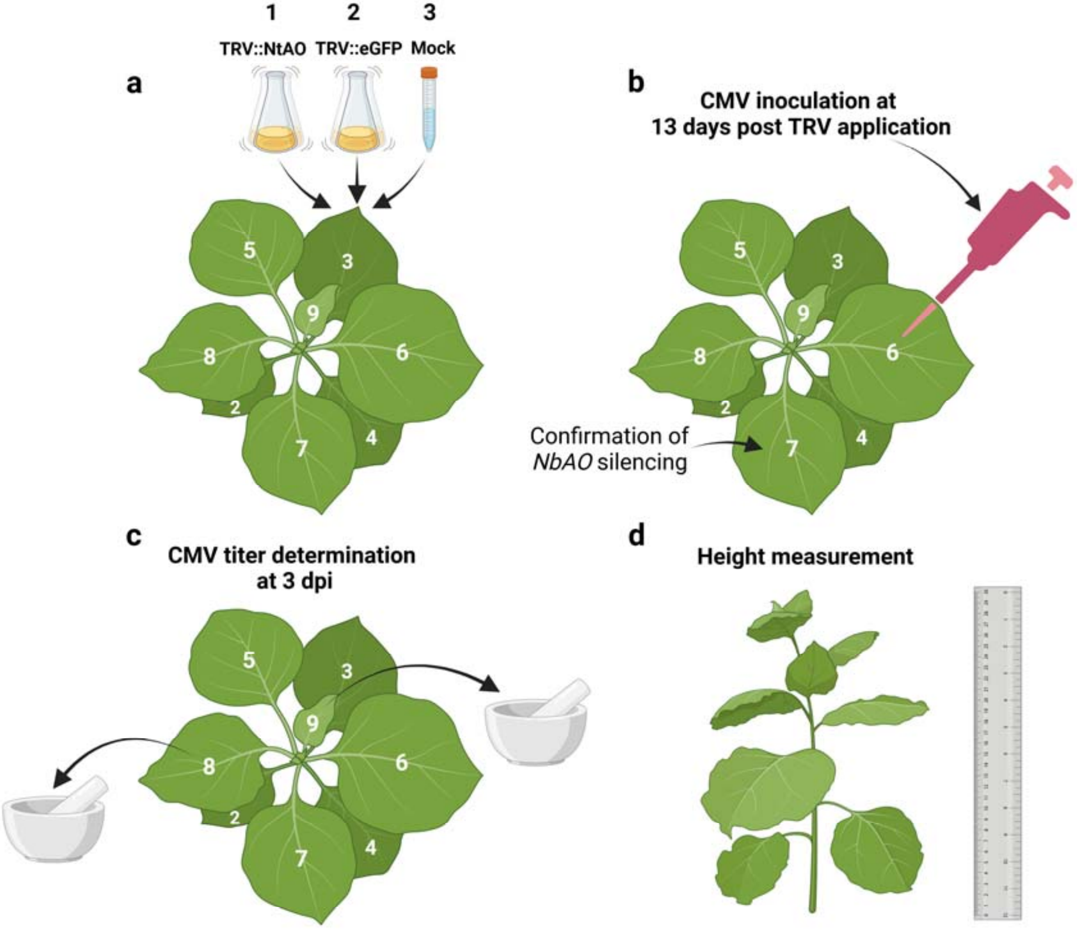

a
1
2
3
TRV::NtAO
TRV::eGFP
Mock
5
3
9
8
6
2
7
4
b
CMV inoculation at
13 days post TRV application
5
3
9
8
6
2
7
4
Confirmation of
NbAO silencing
c
CMV titer determination
at 3 dpi
5
3
9
8
6
2
7
4
d
Height measurement

Fig. S2

a

NbAO_Niben101Scf03026g01009.1

```
MVEHFHQCYLVNFLVLGLLFLTAVSVEARVRHHKWEVKYEYKSPDCFKKLSISINGKTPGPTIVAQQGDTIVVEVKNSLLTENL
AIHWHGIRQPGTYLYHAHYGMQRQAGLQGMIKVLVPDGVVEPFSYDYDRNILLTDWYHKSTYEQATGLASLPFVWVGEPQSLLI
HGRGMFNCSTPGTDVTLCNATNPQCSPYSMIVIPGKTYRLRIGSLTALSALSFEIEGHNMTVVEADGHYVEPFVVQKLFLYSGE
TYSVLIKADQDPSRNYWASTKVVSRNSATPNGLGIITYYPNHPRRTPPTVPPAGPHWNDVAPRVAQSVAIKSHKDFIHAPPKTS
DRVIVMLNTQNRINGFVRWSVNNVSFNMPHTPYLIALKHNLLHTFEQTPPPENYNHENYDIFSVQKNVNATTSNSIYRLKFNTT
VDIILQNANTMNPNNSETHPWHLHGHDFWVMGYGNGKFNQSIDPKNYNLVNPIMKNTIPVHPYGWTALRFQADNPGVWAFHCHI
ESHFFMGMGVVFEEGIDKVGKLPTSIMGCGDSKKFHRP
```

b

NbAO_Niben101Scf03026g01009.1

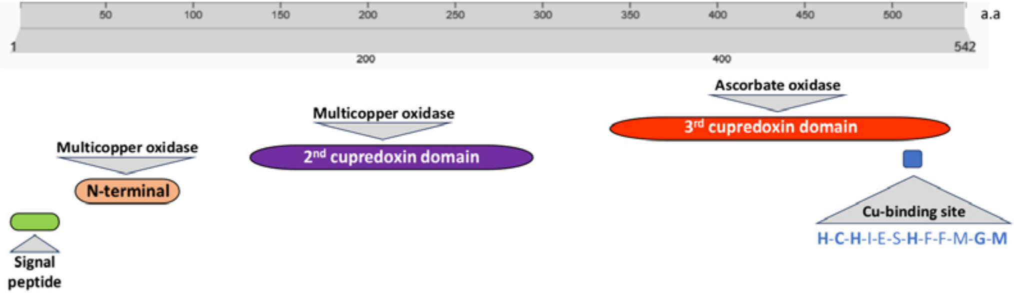

Fig. S3

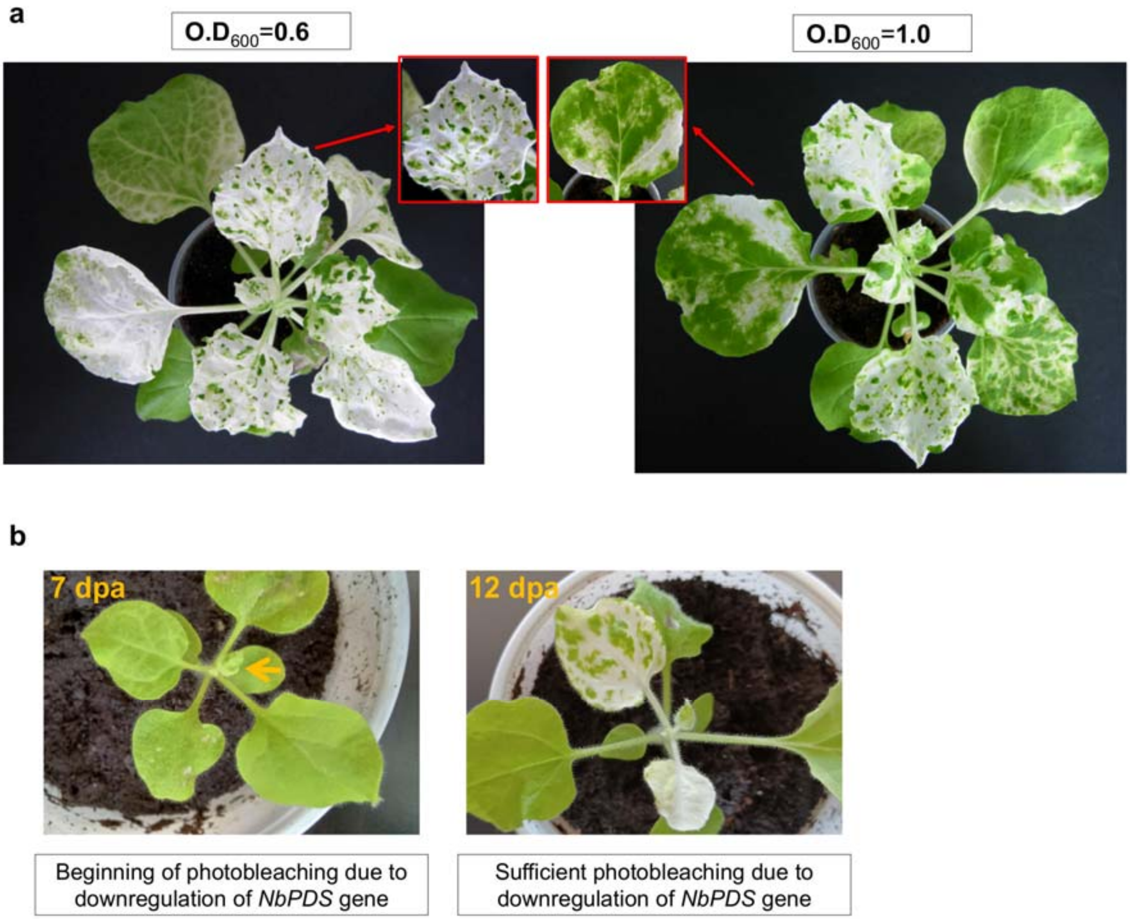
a
O.D600=0.6
O.D600=1.0
b
7 dpa
12 dpa
Beginning of photobleaching due to downregulation of NbPDS gene
Sufficient photobleaching due to downregulation of NbPDS gene

Fig. S4

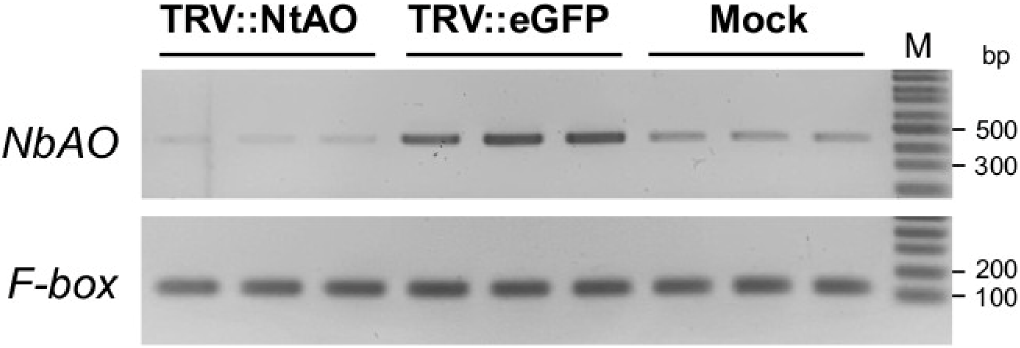

Fig. S5

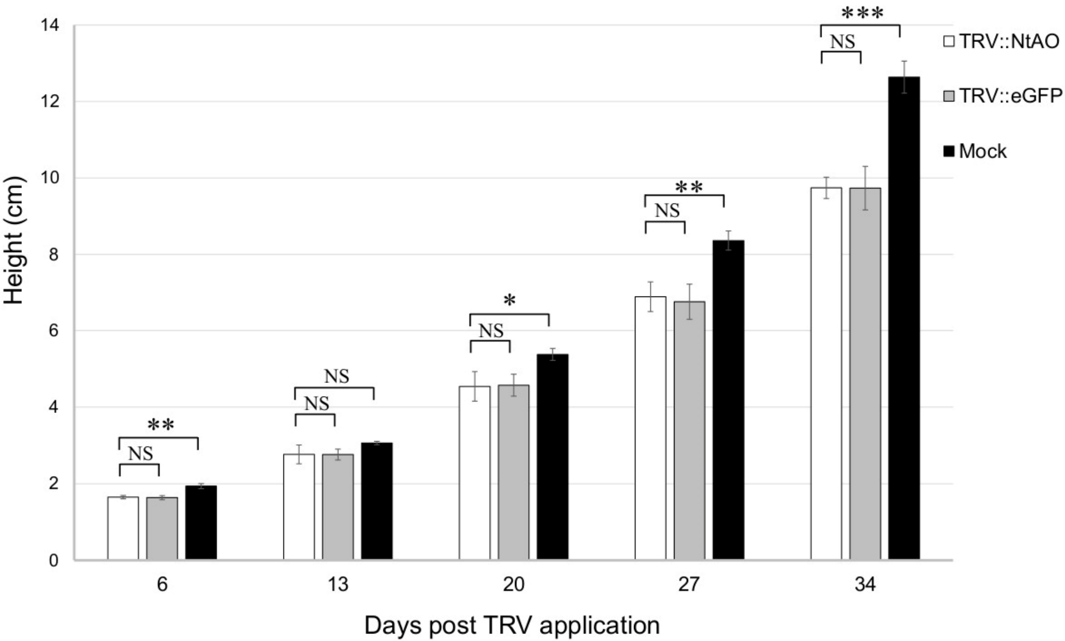
Height (cm)
0
2
4
6
8
10
12
14
6
13
20
27
34
Days post TRV application
TRV::NtAO
TRV::eGFP
Mock
NS
**
NS
NS
NS
*
NS
**
NS
***

Fig. S6

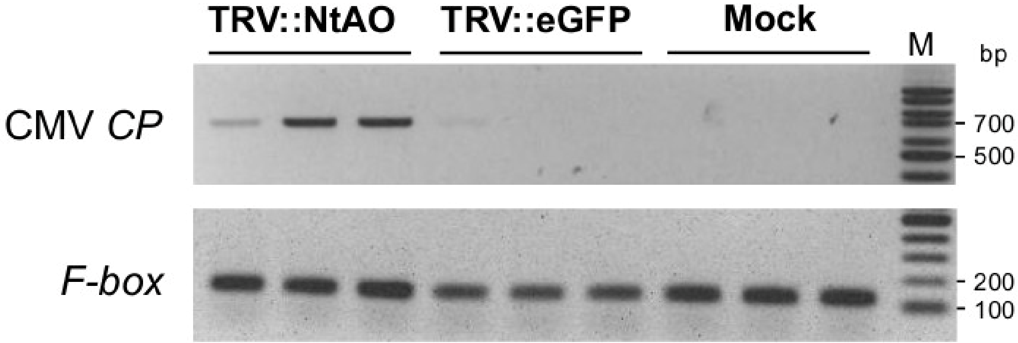

Supplement: Supplementary file 2 — Supplementary file2 (PDF 903 kb) [file 425_2023_4313_MOESM2_ESM.pdf]
